# Informing research design through patient and public involvement; patients and carers with lived experience post-hospital discharge and potential roles for general practice pharmacists

**DOI:** 10.1186/s13104-025-07248-6

**Published:** 2025-04-17

**Authors:** Faiza Yahya, Sam Bartlett, Vibhu Paudyal, Muhammad Abdul Hadi, Hamde Nazar, Ian Maidment

**Affiliations:** 1 Newcastle University, Patient Safety Research Collaboration, School of Pharmacy, King George VI Building, Newcastle-upon-Tyne, NE71RU, UK; 2 University of Birmingham, Edgbaston, B15 2TT Birmingham, UK; 3 Our Health Partnership, B30 3AS Birmingham, UK; 4 PPI contributor, West Midlands, UK; 5 Faculty of Nursing, Midwifery and Palliative Care, King’s College London, WC2R 2LS London, UK; 6 College of Pharmacy, QU Health, Qatar University, Doha, Qatar; 7 School of Pharmacy, College of Health and Life Sciences and Aston Research Centre for Health inAgeing (ARCHA), Aston University, Birmingham, UK

**Keywords:** Patient and public involvement, Post-hospital discharge, Pharmacist, General practice, Medicines management, Transfer of care

## Abstract

**Background:**

Medication safety across care transitions remains a significant burden on healthcare systems. Patient and Public Involvement (PPI) is useful at the very early stages of intervention development to inform research priorities. The aim of this PPI was to scope patients’ and carers’ lived experiences of medicines management post-hospital discharge to inform the design of a research proposal.

**Methods:**

A research planning PPI workshop and additional one-to-one discussions were undertaken with patients and informal carers who had experienced a recent discharge from hospital and were prescribed regular repeat medications.

**Results:**

The 12 public contributors identified that the priority for patients was not limited to medication management alone but rather a broader care package. Multiple themes as priorities for research emerged: (1) broader holistic and social aspects of care involving various healthcare professionals, (2) practical aspects such as timeliness of follow-up and co-ordination of medication management, and (3) communication with the patient/carer and information transfer between settings.

**Conclusion:**

Valuable insights from this PPI helped inform future research design priorities and identify the need for a more holistic approach to care. Future work with multi-stakeholder engagement involving different professionals across sectors is needed to explore safer integrated transitions of care, as well as the use of ongoing PPI and co-design, considering populations that are most vulnerable.

**Supplementary Information:**

The online version contains supplementary material available at 10.1186/s13104-025-07248-6.

## Background

Evidence has shown that transfer of care between hospital and home can be very problematic, with approximately 50% of adults experiencing medication-related problems at hospital discharge [1]. In the United Kingdom (UK), post-discharge medication-related harm in older adults costs the National Health Service (NHS) an estimated £396 million per year [2], with emergency hospital readmission rates within 30 days of discharge rising year on year [3]. Patient safety across transitions is an area that has been repeatedly identified as an international [4] and UK priority (5–6).

Various transitional care models and the role of primary care or general practice pharmacists post-hospital discharge have been demonstrated outside the UK [7–10]. These have identified key components in effective post-discharge care, such as medicine reconciliation, electronic tools, and discharge planning [8]. Most interventions have been targeted at frail older adults^2,7–8,12−13^, who are deemed at higher risk of hospital admissions [7, 11–13] because of multiple comorbidities and polypharmacy [2, 14]. However, extensive heterogeneity in study designs and interventions is apparent, raising the question of whether this is applicable in the UK healthcare system and all patient cohorts. Guidance and strategies in the UK aimed at improving transitional care have shown that it is important to have fully integrated systems and support structures in place for patients when leaving hospital [7, 15]. However this is variable [6, 15], leaving knowledge gaps to understand the impact on patient and provider outcomes [7].

Patient and Public Involvement (PPI), as defined by the National Institute for Health and Care Research (NIHR), is research done *with* or *by* patients and the public, working collaboratively [16]. Involving public contributors at an early stage of research is key to identifying priorities, ensuring that the research is both relevant and meaningful to end users, gaining expert input from those with lived experience (17–18).

### Aim

The aim of this PPI was to scope patients’ and carers’ lived experiences of medicines management post-hospital discharge to inform the design of a research proposal.

## Methods

### Design and setting

A research planning PPI discussion workshop was arranged at a local community venue in Birmingham, United Kingdom. Patients or their carers who were recently discharged from a hospital (within the last 12 months) and were prescribed regular repeat medications were invited to participate.

An advertisement (Appendix 1) was shared by the local patient participation groups (PPG) in their respective local primary care network (PCN). It was also displayed in local GP practice waiting areas, local pharmacies, local community centres and shared on Healthwatch Birmingham to support diversity and inclusion. Eligible patients were also emailed the advert with verbal consent after a post-hospital discharge medication review.

A PPI workshop was convened to discuss the proposed research with the public contributors. A plain English summary of the proposed research area was verbally presented to the attendees before discussions. The focus was to allow patients and informal caregivers to express and share their lived experiences post-hospital discharge in relation to medication management. In addition, the PPI sought to explore the potential role of general practice pharmacists post-hospital discharge and role in future research. A discussion guide was used to support discussions (Appendix 2). During the face-to-face workshop, discussions were audio-recorded with consent from participants and transcribed anonymously by an experienced administrative member of staff.

### Analysis

Suggestions and common understandings from the PPI discussions were collated to generate themes using a thematic analysis approach to find common patterns that would guide a future research focus [19]. A summary of results was sent to the contributors as an opportunity to feedback and co-create themes. (Table 1) These were then reported using the internationally recognised GRIPP2- short form for reporting Patient and Public Involvement [20].

## Results

A total of 12 public contributors participated (eight females and four males of five various ethnic groups and ages ranging from late 30’s to 80’s). Eight participants attended the one-hour PPI group workshop, whereas four contributors preferred a one-to-one discussion by telephone. Where an interpreter was required, a healthcare professional fluent in the native language facilitated.

## Key themes

**Table 1:** Summary of key research themes identified

Priority themes	Research considerations identified
**Broader social aspects**	Which health and social care professionals are involved in the hospital discharge process and the need to interview a range of care professionals with these roles?
**Practical Aspects**	
1. Timeliness of follow-up after hospital discharge	What is the ideal time post-hospital discharge for implementing an intervention?
2. Co-ordination of medicines continuity	What are the key issues that patients face with medication continuity? Is there a standardised pathway for information transfer and medicines supply in the community post-discharge? Exploring process-mapping of current pathways?
**Communication**	
1. To the patient and education on their medicines and condition	What is the impact of communication with patients post-hospital discharge? The research should look to recruit patients from both the hospital setting (prior to discharge) and the general practice setting (post-discharge) Interviews with patients are useful as well as multi-stakeholder engagement with different professionals, agencies and informal carers. Interviewing family members or children (young carers) of those who live alone and require support for healthcare management.
2. Between health care professionals	What systems are in place to enable better transfer of information across settings and how efficient/timely are these systems? How do we use digital technology to improve timeliness of care post-hospital discharge? Multi-stakeholder engagement is important to identify what issues and barriers can impact communication across sectors. Experience-based co-design of an intervention was identified as a potential method.
**Populations to focus research (highest risk)**	Disadvantaged/vulnerable patients; identified as those who were socially isolated, lacked understanding or had language barriers, including but not limited to older adults. What processes are in place to identify and support these patients? The need to identify the role of discharge co-ordinators in the transition process across sectors, i.e. in hospital and after hospital in the community.

### Broader social aspects as a priority for research

While the initial focus of the PPI was on issues related to medication post-hospital discharge and the role of general practice pharmacists in ensuring medication continuity, it quickly became evident that patients’ concerns extended beyond medications alone. This shift in perspective acknowledged a more holistic view of follow-up and communication processes as important to patients at a vulnerable time. The discussions acknowledged the evolving roles of pharmacists within general practice [21], but lacked awareness of how they are actively supporting patients post-discharge. Conversations highlighted the necessity of involving various healthcare professionals in future research, spanning both the secondary and primary care sectors, and exploring opportunities for integration in the transition process.

### Practical aspects as a priority for research

#### Timeliness of follow-up after hospital discharge

The timeliness of follow-ups was identified by most public contributors as a major factor for medication safety. Discussions encompassed who should be responsible for conducting the follow-up in a proposed research intervention and the appropriate timing. Although it was assumed that it would be the GP, acknowledgement was given that other healthcare professionals in primary care could meet the patients’ health and social care requirements.

Perspectives for ideal timeliness varied among public contributors, suggesting opinions between 12 h and 14 days post-discharge. This highlighted that further research is needed to identify the ideal time for follow-up. Concerns were raised about medication discrepancies not being noticed ‘quick enough’, particularly if these were high-risk drugs that required close monitoring (I.e. warfarin), reinforcing the need for timely and accurate information transfer.

#### Continuity and co-ordination of medication management

Contributors mentioned that patients may struggle with the coordination of medicine supplies after being discharged from hospital, especially if there are changes or contraindications of medications. Therefore, the need to explore timely continuity and co-ordination is important, recognising that this has potentially different levels of risk to patients.

### Communication

The theme of communication was prominent in all discussions, both relating to communication with the patient and/or informal carer themselves and between health care professionals.

#### Communication with the patient and education on their medicines and condition

The public contributors highlighted that clear communication to the patient and/or carer was important to them, and research into how a pharmacist’s role may help patients understand and take ownership of their condition needs further exploration. Discussions reiterated that interaction with a pharmacist on the ward before discharge was not common and was also dependent on the time of day the patient was discharged (i.e. out of hours) or by whom. All public contributors suggested that the proposed research should explore current pathways by looking at streamlined processes, considering different locations and timings.

#### Between health care professionals

The public contributors highlighted that there were often problems with communication between sectors (hospitals and the GP practice) and within sectors (hospitals within the same trust) when patients are transferred between settings, and there is a need to explore this further. This disjointed care made public contributors feel that follow-up discussions with the GP and decision-making were difficult. It was agreed that digital technology has a role in safer transfer of information but needs to be explored further as there were concerns that different settings that share the care for a patient do not always exchange information effectively, making the continuity of care for a patient difficult and frustrating. Most public contributors felt that the planned research should explore the communication and follow-up mechanisms post-discharge, especially for those more vulnerable who may struggle with co-ordination.

### Comments on knowledge of the role of pharmacists in general practice

In general, there was a lack of awareness of existing services available post-hospital discharge, such as the availability of general practice pharmacists or even the ‘Discharge Medicines service’ which is available in community pharmacies for follow-up medication reviews post-discharge. Public contributors emphasised that signposting to such services should be encouraged, even if by admin staff.

### Views on how vulnerable groups navigate hospital discharge

All the contributors agreed that it would be very difficult for vulnerable groups to deal with changes to their medication and felt that the need for a follow-up or transition of care service in this cohort was important. Most contributors said that they empathise with the elderly, especially those with no support (i.e. live alone) and are deemed vulnerable. In addition, those with compliance aids may have difficulty and need extra support with changes. Several public contributors felt that socially isolated patients could benefit with some transition support such as ‘Home from Hospital’ (A local Harborne-based charity in Birmingham). Currently, these are identified on the ward supported by hospital-based care co-ordinators and support post-discharge with arranging delivery of essentials such as shopping, but it is unclear if they support with medication matters. Other vulnerable groups mentioned were patients with mental health conditions or at risk of suicidal thoughts. Additionally, patients with language barriers may be at risk as the need to ensure comprehension of their medicines was important, albeit if they had family members translating.

In summary, the PPI group suggested that the most disadvantaged or most vulnerable patients that would benefit from such research are those who were socially isolated, lacked understanding, had language barriers, or were elderly; all of whom are perceived at high risk of medication-related problems post-discharge.

### Engaging patient participation in the proposed research

Feedback during discussions regarding how and when to involve patients during the research project recognised that it was important to identify those at high risk before they are discharged (i.e. in the hospital setting) and explore what tools are available to predict risk to tailor the research population. Identifying patients at the point of discharge could be supported by hospital discharge co-ordinators. Furthermore, methods of outreach, i.e., reaching out to patients at clinic appointments, GP surgeries, and hospital or community follow-up events, should also be considered.

### Developing a public involvement group

At the end of the session, an optional invitation was extended to invite contributors to become involved in a PPI advisory group to support the design and delivery of the research project. This would involve activities such as reviewing lay summaries of future proposals, patient involvement in the research objectives, design, and methods (co-design), reviewing interview scripts, and considering challenges for recruitment. Subsequent PPI activities will be costed into the research application, and the PPI advisory group will be offered co-authorship of any resulting publications.

## Discussion

The insights from this PPI helped guide and identify key considerations for the proposed research design, mainly identifying that concerns to patients and carers in the transfer of care process are not solely medication related. Medication-related harm is a significant risk factor; however, recognising that social aspects take precedence in patients’ lives post-hospital discharge and can subsequently impact their ability to manage their medication. The discussion group highlighted a reliance on patients to take ownership of their medication and where there are gaps in the transfer of information between settings [22, 23], relying on patients or family carers being well and able to ‘chase’ medication changes and follow-up.

Patients and carers highlighted the need for research to explore coherent communication and co-ordination post-discharge, especially with medication. This corroborates with previous work [23, 24], but needs to be explored in the advancing integrated care systems. Despite ongoing work, risks of harm to patients from medicine mismanagement, suboptimal care, and hospital readmissions remain [25].

Some studies aimed at improving coordination between secondary and primary care have explored conceptual maps of patient preferences [26]. However, awareness of professional roles that can support this and interoperability of clinical systems remain challenges (27–28) and the PPI workshop highlighted the importance of exploring this.

To our knowledge, this is the first published PPI that considers medication management post-hospital discharge, especially in the context of pharmacists in general practice. Giving patients and/or carers the opportunity to discuss their lived experiences initially through listening and discussion supports the implementation of co-design during research development and partnerships [29]. This can also complement methods such as qualitative research in the inception and implementation of trials [30]. The increasing use of PPI in general practice research emphasises the importance of transparency and clear reporting (31–32), as well as suitability of the public contributors and preparedness of the researcher to ensure that the PPI is meaningful [33].

### Strengths and limitations

Strengths included diversity within the group and complements a recent scoping review of literature [10], giving first-hand patient perspectives to guide research priorities. The broad topic nature meant that occasionally, discussions steered off-topic and needed to be guided back to the main aim. Considering that this was PPI and not a research study, involving a small number of public contributors, saturation may not have been reached and there may be other perspectives unidentified within this cohort or applicable in different geographical locations and care systems.

## Conclusions and future work

This PPI encourages a broader holistic research design focus, emphasising the need to look at communication and integration across settings, involving various healthcare professionals rather than looking at one healthcare role in isolation or a solely medicines-focus. Further refinement with multi-stakeholder engagement will allow us to understand key factors in successful integrated care pathways, including barriers, enablers, and any environmental constraints, to bridge gaps in care.

With digital advancements, we could better understand how the use of technology to improve timeliness, co-ordination and continuity should be used, ensuring that those who are vulnerable are supported.

## Electronic supplementary material

Below is the link to the electronic supplementary material.

Supplementary Material 1

Supplementary Material 2

Supplementary Material 3

## Data Availability

The datasets generated and/or analysed during the current study are not publicly available due in line with the principles of patient and public involvement not requiring ethical approval or written consent from the public contributors for data to be shared publicly. If required, specific data however may be available from the corresponding author on reasonable request and with permission of the public contributors involved.

## Abbreviations

PPI: Patient and Public Involvement
UK: United Kingdom
GP: General Practitioner
NHS: National Health Service
NIHR: National Institute of Health and Care Research
RDS: Research Design Service
PCN: Primary Care Network
PPG: Patient Participation Group
PALS: Patient and liaison services
GRIPP: Guidance for Reporting Involvement of Patients and the Public
