# Correction: Informing research design through patient and public involvement; patients and carers with lived experience post-hospital discharge and potential roles for general practice pharmacists

**DOI:** 10.1186/s13104-025-07311-2

**Published:** 2025-07-10

**Authors:** Faiza Yahya, Sam Bartlett, Vibhu Paudyal, Muhammad Abdul Hadi, Hamde Nazar, Ian Maidment

**Affiliations:** 1 Newcastle University, Patient Safety Research Collaboration, School of Pharmacy, King George VI Building, Newcastle-upon-Tyne, NE71RU, UK; 2 University of Birmingham, Edgbaston, B15 2TT Birmingham, UK; 3 Our Health Partnership, B30 3AS Birmingham, UK; 4 PPI contributor, West Midlands, UK; 5 Faculty of Nursing, Midwifery and Palliative Care, King’s College London, WC2R 2LS London, UK; 6 College of Pharmacy, QU Health, Qatar University, Doha, Qatar; 7 School of Pharmacy, College of Health and Life Sciences and Aston Research Centre for Health inAgeing (ARCHA), Aston University, Birmingham, UK

**Correction: BMC Research Notes (2025) 18:181**

10.1186/s13104-025-07248-6

Following publication of the original article [1] we became aware that the affiliations of co-authors Vibhu Paudyal and Muhammad Abdul Hadi were unfortunately interchanged.

Dr. Vibhu Paudyal is affiliated with the Faculty of Nursing, Midwifery and Palliative Care, King’s College London, London, WC2R 2LS, UK. Dr. Muhammad Abdul Hadi is affiliated with the College of Pharmacy, QU Health, Qatar University, Doha, Qatar.

The correct authors’ affiliations are shown in this Correction.

The original article has been corrected.

