# Supplementary material for: Informing research design through patient and public involvement; patients and carers with lived experience post-hospital discharge and potential roles for general practice pharmacists
Source: BMC Res Notes. 2025 Apr 17;18:181. doi: 10.1186/s13104-025-07248-6 (PMC12007321; doi:10.1186/s13104-025-07248-6)
Supplement: Supplementary file 1 — Supplementary Material 1 [file 13104_2025_7248_MOESM1_ESM.pptx]

## Slide 1
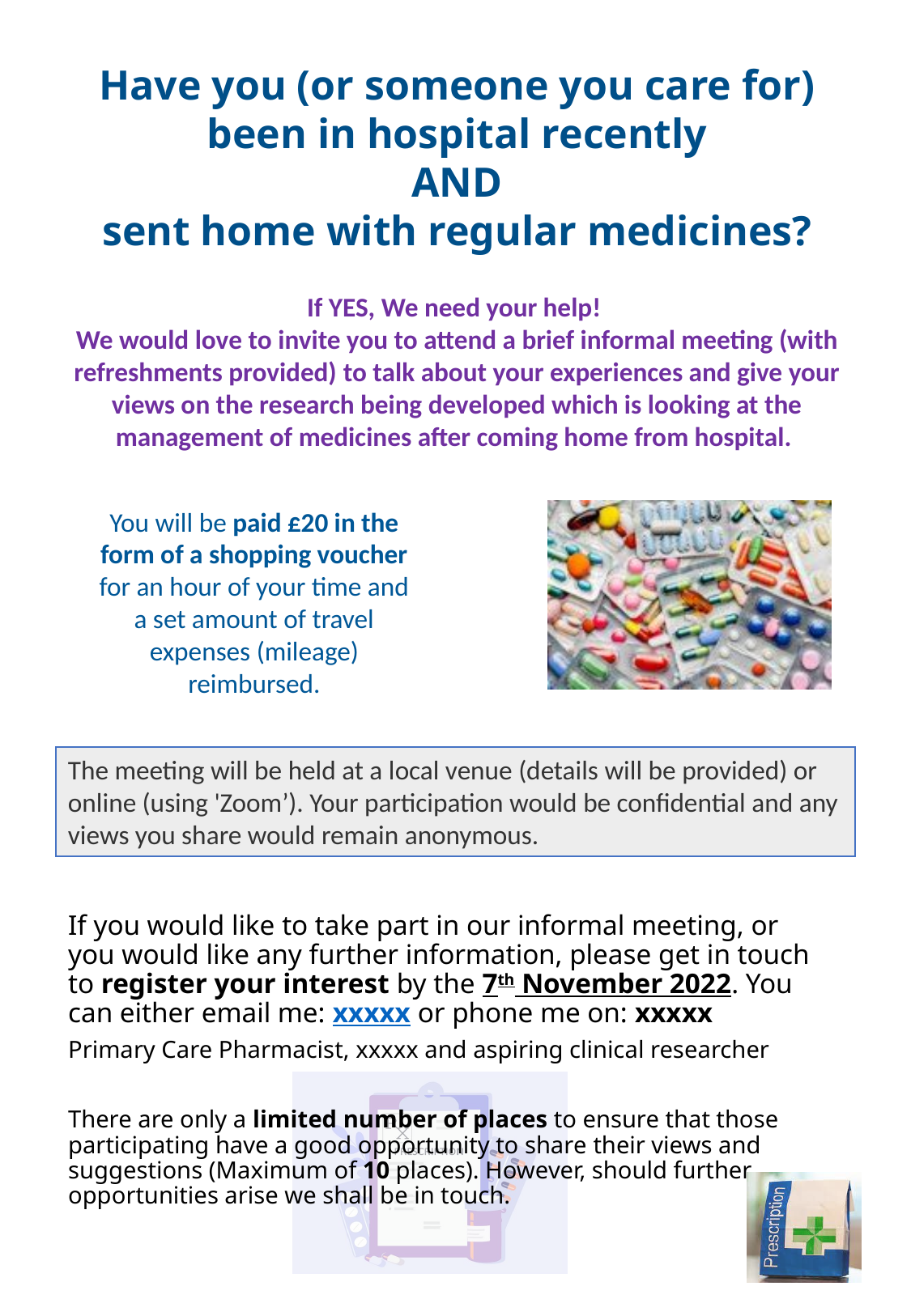

​Have you (or someone you care for)​
been in hospital recently​
AND​
sent home with regular medicines?​
If YES, ​We need your help!
We would love ​to invite you to attend a brief informal meeting (with refreshments provided) to talk about your experiences and give your views on the research being developed which is looking at the management of medicines after coming home from hospital.
You will be paid £20 in the form of a shopping voucher for an hour of your time and a set amount of travel expenses (mileage) reimbursed.​
The meeting will be held at a local venue (details will be provided) or online (using 'Zoom’). Your participation would be confidential and any views you share would remain anonymous.
If you would like to take part in our informal meeting, or you would like any further information, please get in touch to register your interest by the 7th November 2022. You can either email me: xxxxx or phone me on: xxxxx
Primary Care Pharmacist, xxxxx and aspiring clinical researcher
There are only a limited number of places to ensure that those participating have a good opportunity to share their views and suggestions (Maximum of 10 places). However, should further opportunities arise we shall be in touch.
