# Supplementary material for: Informing research design through patient and public involvement; patients and carers with lived experience post-hospital discharge and potential roles for general practice pharmacists
Source: BMC Res Notes. 2025 Apr 17;18:181. doi: 10.1186/s13104-025-07248-6 (PMC12007321; doi:10.1186/s13104-025-07248-6)
Supplement: Supplementary file 2 — Supplementary Material 2 [file 13104_2025_7248_MOESM2_ESM.docx]

**Appendix 2. Discussion Guide**

**Discussion Guide**

The discussions were tailored around these key areas:

1. Project Background, Aims/Objectives of PPI - Lay Summary
2. In relation to medicines, Participants were given the opportunity to speak about their recent experience (or anyone they have cared for) after coming out of hospital.
3. What they think care after coming out of hospital should look like and what would have made their experience better.
4. Defining what patients/carers felt was important to them what are the priority areas that the research should focus on.
5. What the important aspects a medication review after coming home from hospital may be able to address and should we consider the ideal timing this review should take place?
6. What type of healthcare professionals may be involved in care post-hospital discharge and who to involve in the research? (Nurses, GPs, Pharmacists (types of pharmacists)
7. To discuss which cohort of patients would benefit most from an intervention post-hospital discharge and should the research focus on a particular group.
8. This would be the first of a series of PPI activity, therefore offer an opportunity to public contributors for expressions of interest for being part of a PPI advisory group for future sessions and continuation of the project.
