# Supplementary material for: Informing research design through patient and public involvement; patients and carers with lived experience post-hospital discharge and potential roles for general practice pharmacists
Source: BMC Res Notes. 2025 Apr 17;18:181. doi: 10.1186/s13104-025-07248-6 (PMC12007321; doi:10.1186/s13104-025-07248-6)
Supplement: Supplementary file 3 — Supplementary Material 3 [file 13104_2025_7248_MOESM3_ESM.docx]

**Table 2. GRIPP2 Short form**

| Section and topic | Item | Reported on page No |
| --- | --- | --- |
| 1: Aim | Report the aim of PPI in the study | 4-5 |
| 2: Methods | Provide a clear description of the methods used for PPI in the study | 6-7 |
| 3: Study results | Outcomes—Report the results of PPI in the study, including both positive and negative outcomes | 7-12 |
| 4: Discussion and conclusions | Outcomes—Comment on the extent to which PPI influenced the study overall. Describe positive and negative effects | 12-14 |
| 5: Reflections/critical perspective | Comment critically on the study, reflecting on the things that went well and those that did not, so others can learn from this experience | 14 |
